# Clinical implications of peripheral eosinophil count at diagnosis in patients newly diagnosed with microscopic polyangiitis and granulomatosis with polyangiitis

**DOI:** 10.1186/s13075-023-03233-1

**Published:** 2023-12-15

**Authors:** Jang Woo Ha, Sung Soo Ahn, Jason Jungsik Song, Yong-Beom Park, Sang-Won Lee

**Affiliations:** 1 Division of Rheumatology, Department of Internal Medicine, Yonsei University College of Medicine, Seoul, Republic of Korea; 2 Institute for Immunology and Immunological Diseases, Yonsei University College of Medicine, Seoul, Republic of Korea

**Keywords:** Eosinophil, Microscopic polyangiitis, Granulomatosis with polyangiitis, Activity, Mortality

## Abstract

**Background:**

This study investigated the clinical implications of peripheral eosinophil count at diagnosis in estimating cross-sectional antineutrophil cytoplasmic antibody-associated vasculitis (AAV) activity and predicting all-cause mortality during follow-up in patients newly diagnosed with microscopic polyangiitis (MPA) and granulomatosis with polyangiitis (GPA).

**Methods:**

This study included 224 immunosuppressive drug-naïve patients with peripheral eosinophil count at diagnosis < 1,000/mm^3^. The Birmingham Vasculitis Activity Score (BVAS), the Five-Factor Score (FFS), erythrocyte sedimentation rate (ESR) and C-reactive protein (CRP) at diagnosis were assessed.

**Results:**

The median age of the 224 patients (152 MPA and 72 GPA) was 62.0 years; 35.3% of them were men. At diagnosis, peripheral eosinophil count was significantly correlated with BVAS (*P* = 0.001), FFS (*P* = 0.046), ESR (*P* < 0.001), and CRP (*P* < 0.001). Deceased patients had a significantly higher median peripheral eosinophil count at diagnosis than surviving patients (310.0/mm^3^ vs. 170.0/mm^3^, *P* = 0.004). In addition, patients with MPA and those with cardiovascular and renal manifestations at diagnosis exhibited significantly higher peripheral eosinophil counts than those without. When the optimal cut-off of peripheral eosinophil count at diagnosis for all-cause mortality during follow-up was set at 175.0/mm^3^, Patients with peripheral eosinophil count at diagnosis ≥ 175.0/mm^3^ exhibited a significantly lower cumulative patients’ survival rate than those with peripheral eosinophil count at diagnosis < 175.0/mm^3^ (*P* = 0.008).

**Conclusions:**

This study was the first to demonstrate that peripheral eosinophil count at diagnosis could estimate cross-sectional AAV activity at diagnosis and contribute to predicting all-cause mortality during follow-up in MPA and GPA patients.

**Supplementary Information:**

The online version contains supplementary material available at 10.1186/s13075-023-03233-1.

## Background

Antineutrophil cytoplasmic antibody (ANCA)-associated vasculitis (AAV) is characterised by necrotising vasculitis of the small vessels with or without granulomatosis or eosinophil infiltration [1]. AAV includes three subtypes: microscopic polyangiitis (MPA), granulomatosis with polyangiitis (GPA), and eosinophilic granulomatosis with polyangiitis (EGPA) [1, 2]. Among them, an eosinophil-mediated allergic immune mechanism does not play a crucial role in the pathogenesis of MPA and GPA, unlike in EGPA [3, 4]. In addition, in the 2022 American College of Rheumatology (ACR)/ The European Alliance of Associations for Rheumatology classification criteria for MPA and GPA, a negative score is assigned to an item with an increased peripheral eosinophil count [5–7]. Therefore, peripheral eosinophil count does not seem to have a significant clinical implication in patients newly diagnosed with MPA and GPA, versus those with EGPA and allergic diseases [4, 8].

The clinical role of peripheral eosinophil count in the pathogenesis of MPA and GPA has been debated controversially to date: an increased peripheral eosinophil count was reported in the early phases of various acute or chronic diseases other than AAV [9–11]**,** and T_H2_ cell-mediated immune responses and eosinophil activation in the pathogenesis of MPA and GPA were unveiled [12, 13]. Therefore, it could be assumed that peripheral eosinophils may partially or indirectly affect the pathogenesis of MPA and GPA. However, no study has clarified the clinical implications of peripheral eosinophil count at diagnosis in patients newly diagnosed with MPA and GPA in real clinical settings. Hence, in this study, we investigated the clinical implications of peripheral eosinophil count at diagnosis in estimating cross-sectional AAV activity and predicting all-cause mortality during follow-up in patients newly diagnosed with MPA and GPA.

## Methods

### Patients

This study included 224 patients with MPA and GPA who had peripheral eosinophil count at diagnosis < 1,000/mm^3^. They were first diagnosed with MPA or GPA at the authors’ tertiary university hospital from October 2000 to December 2022. In this study, ‘patients newly diagnosed with MPA and GPA’ means ‘patients who were first diagnosed with MPA and GPA at this hospital’. This is because the variables at diagnosis in patients who were diagnosed with MPA and GPA at other hospitals and receiving immunosuppressive treatment are not reliable and do not meet the purpose of this study. All patients fulfilled the diagnostic algorithm for AAV proposed by the European Medicine Agency in 2007, and the revised Chapel Hill Consensus Conference nomenclature of vasculitides proposed in 2012 [1, 2]. They also met the classification criteria for MPA and GPA suggested by a joint group of the American College of Rheumatology and the European Alliance of Associations for Rheumatology in 2022 [5, 6]. Sufficient medical records data were available for each patient for the collection of clinical, laboratory, radiological, and histological variables at diagnosis, as well as death or survival during follow-up. The patients were followed up for more than 3 months after AAV diagnosis, did not have concomitant serious medical conditions mimicking AAV at diagnosis, and had not been exposed to glucocorticoids (≥ 20 mg/day prednisolone equivalent) or immunosuppressive drugs within 4 weeks before AAV diagnosis. The present study was approved by the Institutional Review Board (IRB) of Severance Hospital (Seoul, Korea, IRB No. 4–2020-1071), and was conducted according to the Declaration of Helsinki. Given the retrospective design of the study and the use of anonymised patient data, the requirement for written informed consent was waived.

### Clinical and laboratory data at diagnosis

The variables at diagnosis and during follow-up are described in Table 1. Based on the 2022 classification criteria for MPA and GPA, myeloperoxidase (MPO)-ANCA, and proteinase 3 (PR3)-ANCA measured by immunoassays and perinuclear (P)-ANCA, and cytoplasmic (C)-ANCA detected by indirect fluorescence assays were evenly accepted as ANCA positivity [5, 6]. The Birmingham Vasculitis Activity Score (BVAS) and the Five-Factor Score (FFS) at diagnosis were assessed as AAV-specific indices [14, 15]. Data on type 2 diabetes, hypertension, and dyslipidaemia at diagnosis were collected as the initial comorbidities as well as the traditional risk factors for all-cause mortality in addition to age, male sex, body mass index (BMI), and ex-smoker status [16].

**Table 1:** Characteristics of patients with MPA and GPA (*N* = 224)

Variables	Values	Variables	Values
***At AAV diagnosis***		***During AAV follow-up***	
**Demographic data**		**Poor outcomes (N, (%))**	
Age (years)	62.0 (50.0–70.0)	All-cause mortality	36 (16.1)
Male sex (*N*, (%))	79 (35.3)	Relapse	71 (31.7)
BMI (kg/m^2^)	22.7 (20.5–24.6)	ESKD	53 (23.7)
Ex-smoker (*N*, (%))	6 (2.7)	CVA	17 (7.6)
		ACS	7 (3.1)
**AAV subtype (*N*, (%))**			
MPA	152 (67.9)	**Follow-up duration based on each poor outcomes (months)**	
GPA	72 (32.1)	All-cause mortality	47.8 (12.6–77.5)
**ANCA type and positivity (*N*, (%))**		Relapse	23.2 (6.1–57.7)
MPO-ANCA (or P-ANCA) positivity	164 (73.2)	ESKD	36.0 (6.8–69.6)
PR3-ANCA (or C-ANCA) positivity	41 (18.3)	CVA	40.1 (10.2–75.4)
**AAV-specific indices**		ACS	46.5 (12.0–77.1)
BVAS	12.0 (6.3–18.0)	**Medications (*N*, (%))**	
FFS	1.0 (0–2.0)	Glucocorticoids	208 (92.9)
**Comorbidities (*N*, (%))**		Cyclophosphamide	122 (54.5)
T2DM	65 (29.0)	Rituximab	44 (19.6)
Hypertension	91 (40.6)	Mycophenolate mofetil	49 (21.9)
Dyslipidaemia	47 (21.0)	Azathioprine	114 (50.9)
**Laboratory results**		Tacrolimus	22 (9.8)
White blood cell count (/mm^3^)	8,825.0 (6,385.0–12,322.5)	Methotrexate	23 (10.3)
Neutrophil count (/mm^3^)	6,765.0 (4,335.0–10.155.0)		
Lymphocyte count (/mm^3^)	1,345.0 (932.5–1,867.5)		
Monocyte count (/mm^3^)	450.0 (330.0–597.5)		
Basophil count (/mm^3^)	20.0 (10.0–40.0)		
Haemoglobin (g/dL)	10.9 (9.3–12.8)		
Platelet count (× 1000/mm^3^)	292.0 (223.0–393.8)		
Fasting glucose (mg/dL)	99.0 (90.0–127.0)		
Blood urea nitrogen (mg/dL)	18.4 (13.9–36.6)		
Serum creatinine (mg/dL)	1.0 (0.7–2.1)		
Serum total protein (g/dL)	6.7 (6.0–7.2)		
Serum albumin (g/dL)	3.7 (3.1–4.2)		
ESR (mm/h)	63.0 (23.8–100.3)		
CRP (mg/L)	15.0 (1.7–69.6)		
**Eosinophil count (/mm**^**3**^**)**	190.0 (72.5–360.0)		

*MPA* Microscopic polyangiitis, *GPA* Granulomatosis with polyangiitis, *BMI* Body mass index, *MPO* Myeloperoxidase, *ANCA* Antineutrophil cytoplasmic antibody, *P* Perinuclear, *PR3* Proteinase 3, *C* Cytoplasmic, *BVAS* Birmingham vasculitis activity score, *FFS* Five-factor score, *T2DM* Type 2 diabetes mellitus, *ESR* Erythrocyte sedimentation rate, *CRP* C-reactive protein, *ESKD* End-stage kidney disease, *CVA* Cerebrovascular accident, *ACS* Acute coronary syndrome

Values are expressed as a median (25th-75th percentile) or *N* (%)

### Poor outcomes and follow-up duration

We investigated the incidence of all-cause mortality, relapse, end-stage kidney disease (ESKD), cerebrovascular accident (CVA), and acute coronary syndrome (ACS) during follow-up as poor MPA and GPA outcomes. In terms of the follow-up duration based on each poor outcome, it was defined as the period from diagnosis to each poor outcome in patients with each poor outcome. Whereas, it was defined as the period from diagnosis to the last visit in patients without each poor outcome. We counted the number of patients who received glucocorticoids, cyclophosphamide, rituximab, mycophenolate mofetil, azathioprine, tacrolimus, and methotrexate during follow-up after diagnosis.

### Statistical analyses

All statistical analyses were performed using SPSS Statistics for Windows, version 26 (IBM Corp., Armonk, NY, USA). Continuous variables are expressed as medians (25th-75th percentiles), whereas categorical variables are expressed as numbers (percentages). The correlation coefficient (r) between the two variables was obtained using the Pearson correlation analysis. Significant differences between two continuous variables were compared using the Mann–Whitney U test. The optimal cut-off was extrapolated by the receiver operating characteristic (ROC) curve analysis and one value having the maximised sum of sensitivity and specificity. Comparison of the cumulative survivals rates between the two groups was analysed by the Kaplan–Meier survival analysis with the log-rank test. The multivariable Cox hazard model using variables with statistical significance in the univariable Cox hazard model was conducted to appropriately obtain the hazard ratios (HRs) during the considerable follow-up duration. *P* < 0.05 were considered statistically significant.

## Results

### Characteristics

At diagnosis, the median age of the 224 patients (152 MPA and 72 GPA) was 62.0 years; 35.3% of them were men. MPO-ANCA (or P-ANCA), and PR3-ANCA (or C-ANCA) were detected in 164 and 41 patients, respectively. The median BVAS, FFS, erythrocyte sedimentation rate (ESR), and C-reactive protein (CRP) levels were 12.0, 1.0, 63.0 mm/h, and 15.0 mg/L, respectively. The median peripheral eosinophil count was 190.0/mm^3^. During follow-up, 36 patients died for a median follow-up duration based on all-cause mortality of 47.8 months. Of the 152 and 72 patients with MPA and GPA, 24 and 12 died, respectively, and there was no significant difference in the mortality rates between the two groups (15.8% vs. 16.7%, *P* = 0.867). Overall, 71, 53, 17, and seven patients experienced relapse, ESKD, CVA, and ACS, respectively. Glucocorticoids, cyclophosphamide, and azathioprine were administered to 208, 122, and 114 patients, respectively (Table 1).

### Correlation analysis

**Fig. 1:**
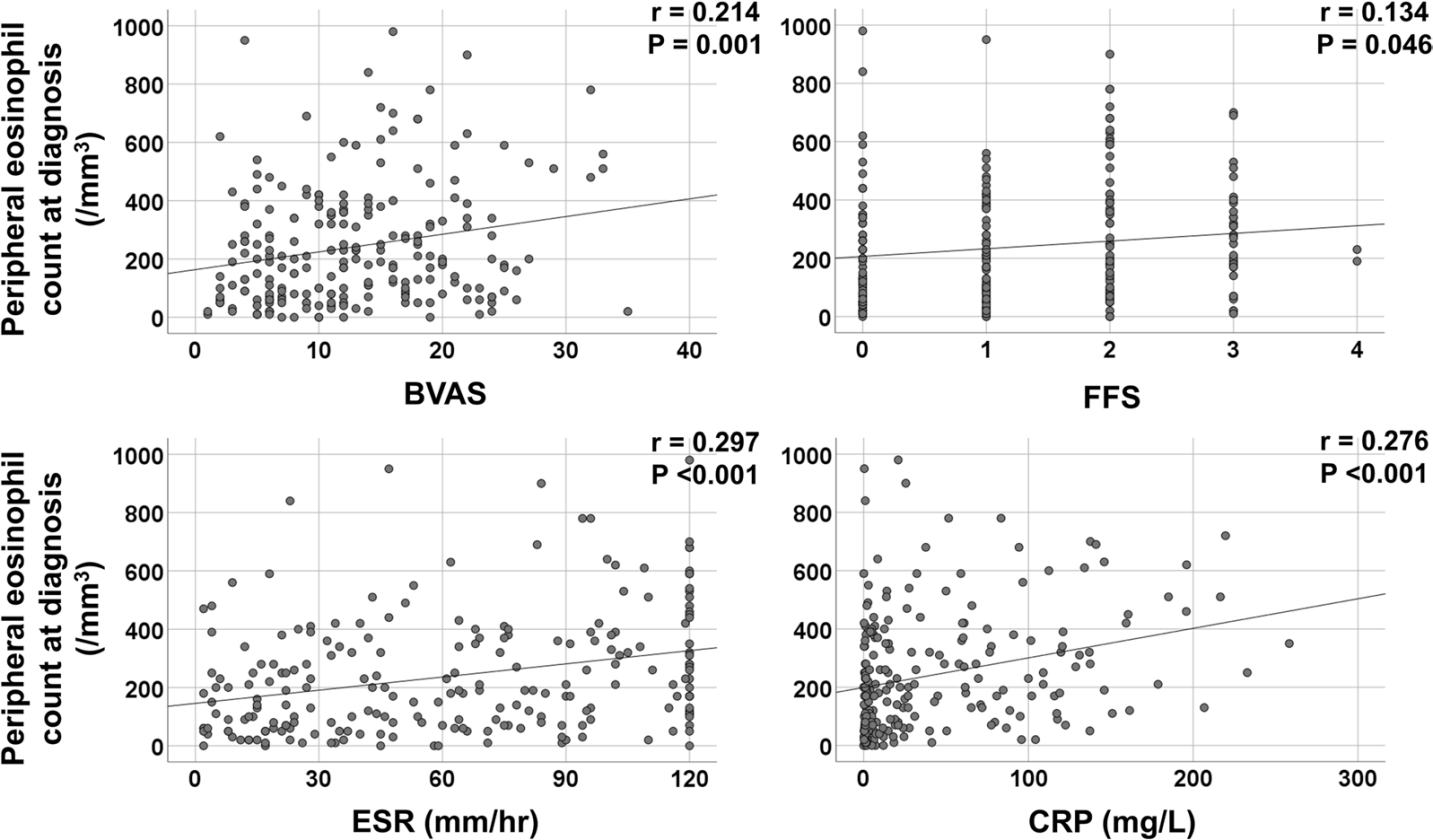
Correlation analysis of peripheral eosinophil count with BVAS, FFS, ESR, and CRP at diagnosis. *BVAS* the Birmingham vasculitis activity score, *FFS* the five-factor score, *ESR* Erythrocyte sedimentation rate, *CRP* C-reactive protein

At diagnosis, peripheral eosinophil count was significantly correlated with BVAS (*r* = 0.214, *P* = 0.001), FFS (*r* = 0.134, *P* = 0.046), ESR (*r* = 0.297, *P* < 0.001), and CRP (*r* = 0.276, *P* < 0.001) (Fig. 1).

### Comparison analysis

**Fig. 2:**
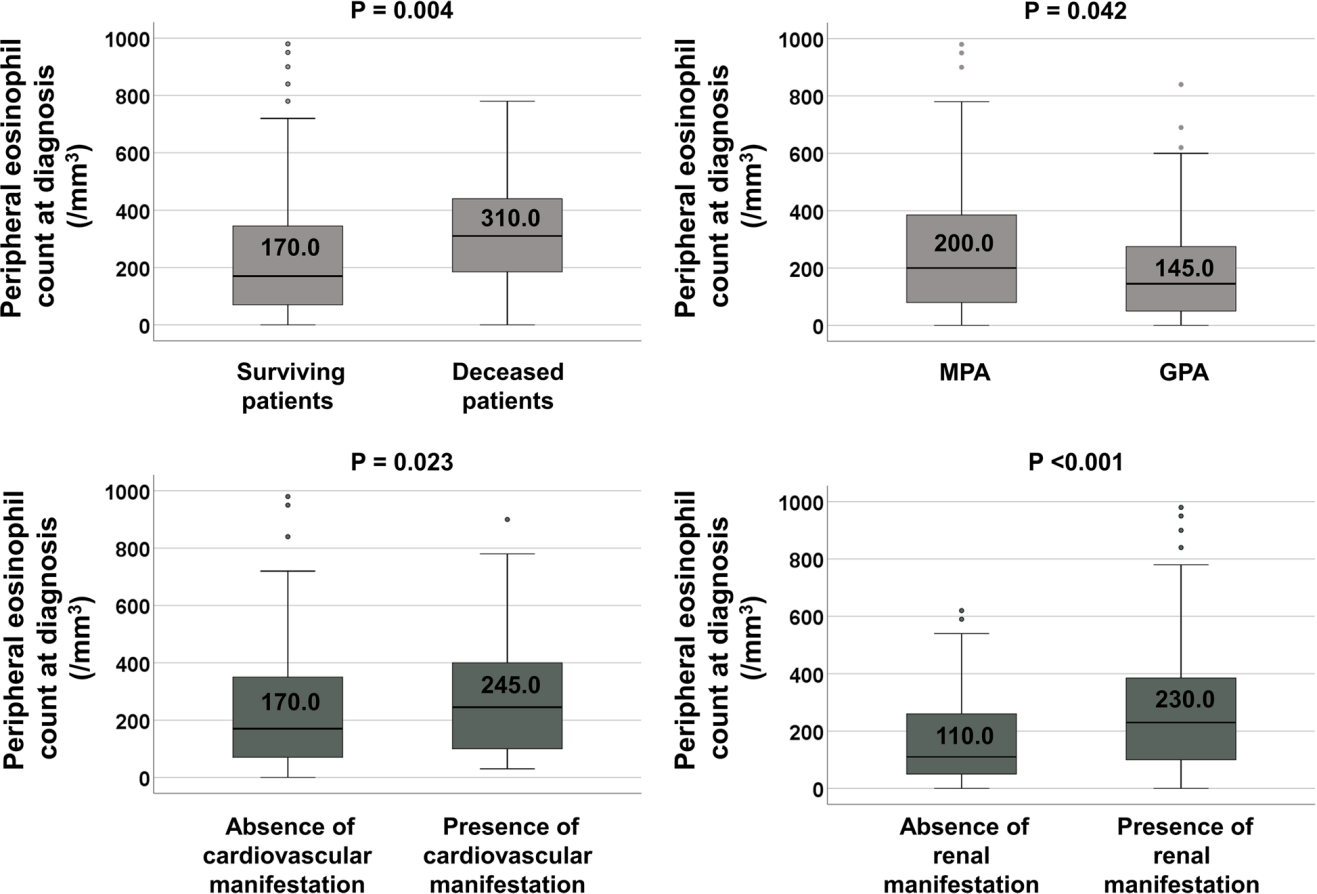
Comparison analysis of peripheral eosinophil counts according to death during follow-up, MPA or GPA, and clinical manifestations at diagnosis. *MPA* Microscopic polyangiitis, *GPA* Granulomatosis with polyangiitis

Deceased patients had a significantly higher median peripheral eosinophil count at diagnosis than surviving patients (310.0/mm^3^ vs. 170.0/mm^3^, *P* = 0.004). At diagnosis, the median peripheral eosinophil count was significantly higher in MPA patients than in GPA patients (200.0/mm^3^ vs. 145.0/mm^3^, *P* = 0.042). However, no significant differences in peripheral eosinophil counts were observed with respect to sex, MPO-ANCA (or P-ANCA) positivity, or PR3-ANCA (or C-ANCA) positivity. On the other hand, among the BVAS items, patients with cardiovascular and renal manifestations at diagnosis exhibited significantly higher peripheral eosinophil counts than those without them (245.0/mm^3^ vs. 170.0/mm^3^, *P* = 0.023, and 230.0/mm^3^ vs. 110.0/mm^3^, *P* < 0.001). (Fig. 2).

### Optimal cut-off

**Fig. 3:**
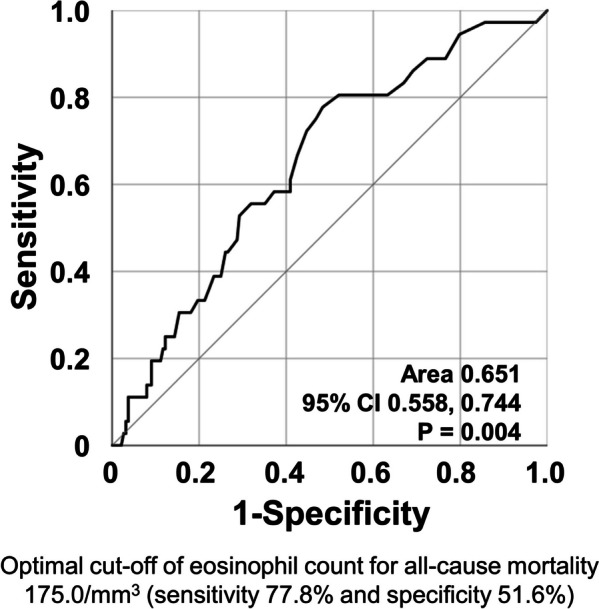
ROC curve analysis for the optimal cut-off of peripheral eosinophil count at diagnosis for all-cause mortality during follow-up. *ROC* the receiver operating characteristic

Using the ROC curve, when the optimal cut-off of peripheral eosinophil count at diagnosis for all-cause mortality during follow-up was set at 175.0/mm^3^, the sensitivity, and specificity were 77.8% and 51.6%, respectively (Fig. 3).

### Comparison of cumulative patients’ survival rates

**Fig. 4:**
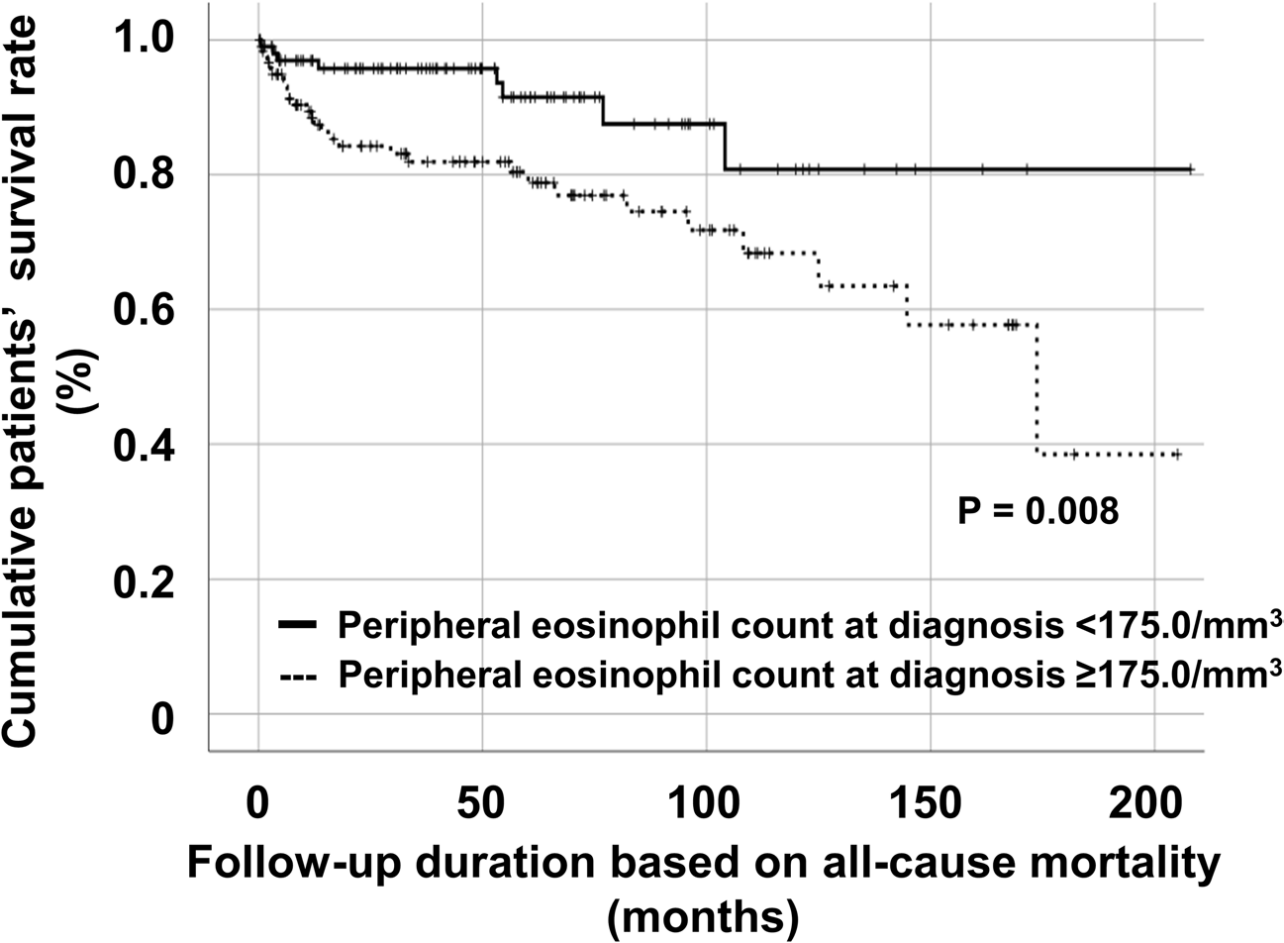
Comparison of cumulative patients’ survival rates according to peripheral eosinophil count of 175/mm.^3^

Patients with peripheral eosinophil count at diagnosis ≥ 175.0/mm^3^ exhibited a significantly lower cumulative patients’ survival rate than those with peripheral eosinophil count at diagnosis < 175.0/mm^3^ (*P* = 0.008) (Fig. 4).

## Discussion

Among the three AAV subtypes, eosinophils are considered more closely associated with EGPA occurrence than with MPA and GPA [4]. According to the new classification criteria for AAV announced in 2022, + 5 points were assigned to an item of peripheral eosinophil count at diagnosis ≥ 1,000/mm^3^ in the 2022 classification criteria for EGPA, versus, -4 points in the criteria for MPA and GPA [5, 16, 17]. Additionally, according to the treatment guidelines for AAV presented by the ACR in 2021, mepolizumab, which inhibits the signal transduction of interleukin-5, a cytokine closely related to eosinophils only in the treatment algorithm for EGPA, is recommended, unlike MPA and GPA [18]. Therefore, an increase in peripheral eosinophil count in patients newly diagnosed with MPA and GPA might be negligible; however, this study’s findings have the clinical implications.

A previous study reported results contrary to ours including a decrease in peripheral eosinophil count and the expression of altered surface markers of eosinophils in MPA and GPA patients versus healthy controls. However, the most significant difference between the previous study and our study was the use of glucocorticoids and immunosuppressive drugs. Because the previous study included patients taking therapeutic drugs, it was difficult to rule out the possibility that those drugs might have an influence on decreased peripheral eosinophil count in the group of MPA and GPA patients [19]. However, because our study included only patients who had not been exposed to those drugs within 4 weeks before AAV diagnosis, confounding variables due to glucocorticoids and immunosuppressive drugs might be minimised or nullified. In addition, they did not provide information on the association between peripheral eosinophil count and cross-sectional AAV activity at diagnosis or poor outcomes during follow-up [19]. To overcome these limitations, this study investigated the clinical implications of peripheral eosinophil count at diagnosis in immunosuppressive drug-naïve patients newly diagnosed with MPA and GPA and demonstrated that it could estimate cross-sectional AAV activity and predict all-cause mortality during follow-up.

To investigate the concordance or discordance of the results of the correlation analyses according to AAV subtypes, we performed the correlation analyses separately in patients with MPA and those with GPA. Among the 152 patients with MPA, peripheral eosinophil count was significantly correlated with BVAS (*r* = 0.188, *P* = 0.021), ESR (*r* = 0.333, *P* < 0.001), and CRP (*r* = 0.241, *P* = 0.003) but not FFS. Whereas, among the 72 patients with GPA, peripheral eosinophil count was significantly correlated with only CRP (*r* = 0.372, *P* = 0.001). On the other hand, peripheral eosinophil count tended to be correlated with BVAS but it was not statistically significant (*r* = 0.197, *P* = 0.097). Despite the discordance of the correlation pattern, between MPA and GPA patients, given that in real clinical settings, there are patients classified as both MPA and GPA, patients with unclear boundaries between MPA and GPA, and those whose MPA and GPA diagnoses change over time, we believe that these results may support that peripheral eosinophil count at diagnosis could reflect cross-sectional BVAS and CRP regardless of MPA or GPA patients.

We wondered about the mechanism of the predictive ability of peripheral eosinophil count at diagnosis for all-cause mortality during follow-up in patients newly diagnosed with MPA and GPA, and suggested two hypotheses. The first hypothesis was based on the initial disease activity of MPA and GPA at diagnosis. In this study, we showed that at the time of diagnosis, peripheral eosinophil count was significantly correlated with cross-sectional BVAS and FFS, well-known predictors of all-cause mortality in AAV patients [20, 21]. Moreover, we found that it was significantly correlated with ESR and CRP levels at diagnosis, which could simultaneously reflect the inflammatory burden of AAV [22]. The second hypothesis was related to chemotactic factors of eosinophils. In the pathogenesis of MPA and GPA, interleukin-8, C3a, and C5a are key molecules that can activate and recruit neutrophils to inflamed tissues, leading to a vicious cycle and aggravated disease course [3, 23]. These factors are also known as chemotactic factors for eosinophils [24–26].Therefore, high levels of inflammation that may enhance the production of eosinophil chemotactic factors may subsequently increase not only peripheral eosinophil count but also the likelihood of all-cause mortality.

Another possibility is that the immune properties of EGPA partially contribute to those of MPA and GPA in newly diagnosed patients. In fact, there has been a movement to understand EGPA as a disease that shares parts with GPA, MPA, polyangiitis overlap syndrome, and hypereosinophilic syndrome, rather than as an independent and sequestrated disease [27]. While the result that peripheral eosinophil count was elevated in patients with MPA or renal manifestation may support the two hypotheses mentioned above, the result of the same pattern in patients with cardiovascular manifestation may imply the effect of partially overlapping EGPA (Fig. 2). However, since cardiac magnetic resonance imaging was not performed in all patients with cardiovascular manifestation, and thus, it was impossible to clearly provide objective evidence of eosinophilic myocarditis, we could not insist on this context as a hypothesis; rather, we just suggest its possibility.

Here, we attempted to elucidate the independent predictive potential of peripheral eos2inophil count at diagnosis for all-cause mortality among the variables at diagnosis using the Cox hazards model analyses. In the univariable analysis, age, male sex, BMI, BVAS, FFS, dyslipidaemia, white blood cell count, haemoglobin, blood urea nitrogen, serum creatinine, serum total protein, serum albumin, CRP, and peripheral eosinophil count ≥ 175.0/mm^3^ were significantly associated with all-cause mortality in patients newly diagnosed with MPA and GPA. In contrast, the continuous variable of peripheral eosinophil count was not associated with all-cause mortality (*P* = 0.098).

In the multivariable analysis, male sex (HR 3.463, 95% confidence interval [CI] 1.561, 7.682), BVAS (HR 1.076, 95% CI 1.015, 1.141), FFS (HR 1.560, 95% CI 1.043, 2.332), dyslipidaemia (HR 3.349, 95% CI 1.497, 7.493), and serum albumin (HR 0.377, 95% CI 0.180, 0.789) were independently associated with all-cause mortality in patients newly diagnosed with MPA and GPA, whereas peripheral eosinophil count ≥ 175.0/mm^3^ was not (see Additional file 1). Therefore, it was concluded that peripheral eosinophil count at diagnosis had the ability to predict all-cause mortality during follow-up, but did not have strong predictive potential comparable to those of traditional risk factors in patients newly diagnosed with MPA and GPA.

Given that the normal peripheral eosinophil count is ≤ 500/mm^3^, only 197 patients newly diagnosed with MPA and GPA who had peripheral eosinophil count at diagnosis ≤ 500/mm^3^ were re-analysed. Using the ROC curve, when the optimal cut-off of peripheral eosinophil count at diagnosis for all-cause mortality was set at ≥ 165.0/mm^3^, the sensitivity and specificity were 75.9% and 46.4%, respectively (see Additional file 2A). Patients with peripheral eosinophil count at diagnosis ≥ 165.0/mm^3^ exhibited a significantly reduced cumulative patients’ survival rate compared to those with peripheral eosinophil count at diagnosis < 165.0/mm^3^ (see Additional file 2B). Therefore, regardless of the absolute normal range of peripheral eosinophil count (500/mm3) or the range for EGPA exclusion (1,000/mm3), peripheral eosinophil count at diagnosis showed a pattern similar to the conclusions of this study.

The strength of this study is that it is the first to demonstrate that peripheral eosinophil count at diagnosis can estimate cross-sectional AAV activity and predict all-cause mortality during follow-up in patients newly diagnosed with MPA and GPA. This study has several limitations. The first limitation is the retrospective study design. Because of this study design, we could not completely control for conditions that could cause an increase in peripheral eosinophil count, such as the history of allergic diseases, history of taking medications other than glucocorticoids and immunosuppressive drugs, and the presence or absence of parasitic infections in all included patients. The second limitation is the relatively small number of patients newly diagnosed with MPA and GPA. Because of this limitation, it seems uneasy to generalise the results of this study. The third limitation is not performing validation analysis. Since there are few tertiary hospitals operating the observational and systemic cohorts of Korean patients with AAV except this hospital, it was not possible to perform an additional analysis through a validation cohort considering ethnic and geographical features. Nevertheless, this study has clinical significance in that it unveiled the association of peripheral eosinophil count with AAV activity at diagnosis and all-cause mortality during follow-up in patients newly diagnosed with MPA and GPA as a pilot study. We believe that a future prospective study including more patients will provide more reliable information on the clinical implications of peripheral eosinophil count at diagnosis in patients newly diagnosed with MPA and GPA.

## Conclusions

This study was the first to demonstrate that peripheral eosinophil count at diagnosis could estimate cross-sectional AAV activity at diagnosis and contribute to predicting all-cause mortality during follow-up in patients newly diagnosed with MPA and GPA other than EGPA.

### Supplementary Information

**Additional file 1. **Cox hazards model analyses of eosinophil count and variables at diagnosis for all-cause mortality during follow-up in MPA and GPA patients

**Additional file 2. **ROC curve analysis for the optimal cut-off of peripheral eosinophil count at diagnosis for all-cause mortality during follow-up and comparison of cumulative patients’ survival rates according to peripheral eosinophil count of 165/mm^3^ in MPA and GPA patients who had peripheral eosinophil count at diagnosis≤500/mm^3^

## Data Availability

Additional data are available on request.

## Declarations

### Provenance and peer review

Not commissioned; externally peer reviewed.
