# Supplementary material for: Clinical implications of peripheral eosinophil count at diagnosis in patients newly diagnosed with microscopic polyangiitis and granulomatosis with polyangiitis
Source: Arthritis Res Ther. 2023 Dec 15;25:245. doi: 10.1186/s13075-023-03233-1 (PMC10722771; doi:10.1186/s13075-023-03233-1)
Supplement: Supplementary file 1 — Additional file 1. Cox hazards model analyses of eosinophil count and variables at diagnosis for all-cause mortality during follow-up in MPA and GPA patients [file 13075_2023_3233_MOESM1_ESM.docx]

**Additional file 1. Cox hazards model analyses of eosinophil count and variables at diagnosis for all-cause mortality during follow-up in MPA and GPA patients**

| **Variables** | **Univariable** | | |  | **Multivariable**  **(eosinophil count ≥175.0/mm^3^)** | | |
| --- | --- | --- | --- | --- | --- | --- | --- |
|  | **HR** | **95% CI** | **P value** |  | **HR** | **95% CI** | **P value** |
| Age (years) | 1.057 | 1.024, 1.091 | 0.001 |  | 1.016 | 0.982, 1.051 | 0.353 |
| Male sex (N, (%)) | 2.618 | 1.352, 5.070 | 0.004 |  | 3.463 | 1.561, 7.682 | 0.002 |
| BMI (kg/m^2^) | 1.101 | 1.005, 1.207 | 0.039 |  | 1.062 | 0.952, 1.185 | 0.279 |
| Ex-smoker (N, (%)) | 2.372 | 0.563, 9.992 | 0.239 |  |  |  |  |
| MPA (vs.GPA) | 0.905 | 0.448, 1.825 | 0.780 |  |  |  |  |
| MPO-ANCA (or P-ANCA) positivity | 1.305 | 0.622, 2.738 | 0.481 |  |  |  |  |
| PR3-ANCA (or C-ANCA) positivity | 0.742 | 0.288, 1.913 | 0.537 |  |  |  |  |
| BVAS | 1.078 | 1.034, 1.123 | <0.001 |  | 1.076 | 1.015, 1.141 | 0.014 |
| FFS | 1.930 | 1.380, 2.700 | <0.001 |  | 1.560 | 1.043, 2.332 | 0.030 |
| T2DM | 1.129 | 0.563, 2.264 | 0.732 |  |  |  |  |
| Hypertension | 1.148 | 0.594, 2.219 | 0.682 |  |  |  |  |
| Dyslipidaemia | 2.099 | 1.049, 4.202 | 0.036 |  | 3.349 | 1.497, 7.493 | 0.003 |
| White blood cell count (/mm^3^) | 1.000 | 1.000, 1.000 | 0.001 |  | 1.000 | 1.000, 1.000 | 0.416 |
| Haemoglobin (g/dL) | 0.820 | 0.700, 0.960 | 0.014 |  | 1.093 | 0.876, 1.363 | 0.431 |
| Platelet count (× 1000/mm^3^) | 1.000 | 0.998, 1.003 | 0.665 |  |  |  |  |
| Fasting glucose (mg/dL) | 1.005 | 0.999, 1.011 | 0.083 |  |  |  |  |
| Blood urea nitrogen (mg/dL) | 1.009 | 1.000, 1.017 | 0.043 |  | 0.995 | 0.980, 1.010 | 0.522 |
| Serum creatinine (mg/dL) | 1.143 | 1.006, 1.299 | 0.041 |  | 1.065 | 0.853, 1.329 | 0.579 |
| Serum total protein (g/dL) | 0.527 | 0.350, 0.793 | 0.002 |  | 0.981 | 0.843, 1.142 | 0.805 |
| Serum albumin (g/dL) | 0.361 | 0.229, 0.568 | <0.001 |  | 0.377 | 0.180, 0.789 | 0.010 |
| ESR (mm/hr) | 1.008 | 1.000, 1.017 | 0.056 |  |  |  |  |
| CRP (mg/L) | 1.008 | 1.003, 1.013 | 0.001 |  | 0.997 | 0.990, 1.004 | 0.415 |
| Eosinophil count (/mm^3^) | 1.001 | 1.000, 1.002 | 0.098 |  |  |  |  |
| Eosinophil count ≥175.0/mm^3^ | 2.766 | 1.256, 6.088 | 0.012 |  | 1.337 | 0.578, 3.093 | 0.497 |

MPA: microscopic polyangiitis; GPA: granulomatosis with polyangiitis; BMI: body mass index; MPO: myeloperoxidase; ANCA: antineutrophil cytoplasmic antibody; P: perinuclear; PR3: proteinase 3; C: cytoplasmic; BVAS: Birmingham vasculitis activity score; FFS: five-factor score; T2DM: type 2 diabetes mellitus; ESR: erythrocyte sedimentation rate; CRP: C-reactive protein.
