# Supplementary figures and images for: Clinical implications of peripheral eosinophil count at diagnosis in patients newly diagnosed with microscopic polyangiitis and granulomatosis with polyangiitis
Source: Arthritis Res Ther. 2023 Dec 15;25:245. doi: 10.1186/s13075-023-03233-1 (PMC10722771; doi:10.1186/s13075-023-03233-1)

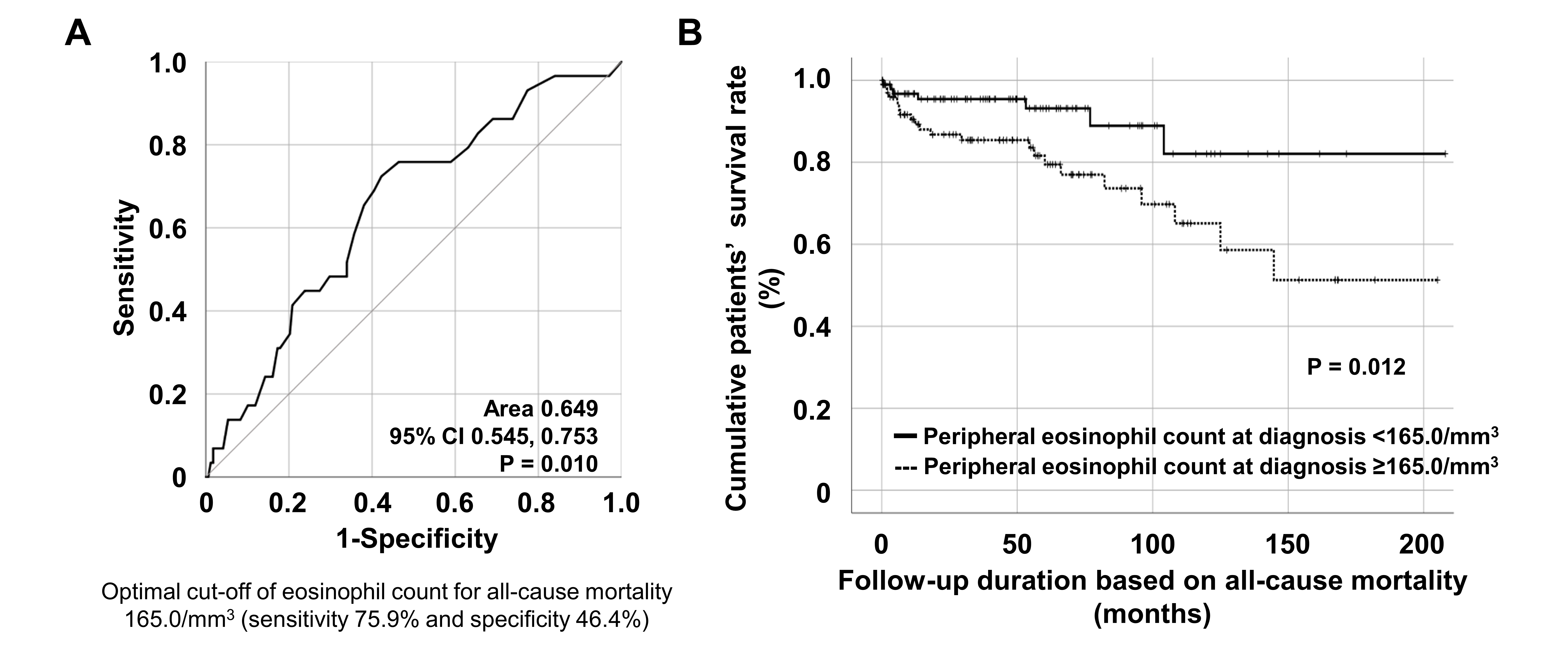

Supplement: Supplementary file 2 — Additional file 2. ROC curve analysis for the optimal cut-off of peripheral eosinophil count at diagnosis for all-cause mortality during follow-up and comparison of cumulative patients’ survival rates according to peripheral eosinophil count of 165/mm3 in MPA and GPA patients who had peripheral eosinophil count at diagnosis≤500/mm3 [file 13075_2023_3233_MOESM2_ESM.tif]
